# CCL3 and MMP-9 are induced by TL1A during death receptor 3 (TNFRSF25)-dependent osteoclast function and systemic bone loss

**DOI:** 10.1016/j.bone.2017.01.002

**Published:** 2017-04

**Authors:** Fraser L. Collins, Jessica O. Williams, Anja C. Bloom, Ravinder K. Singh, Lauren Jordan, Michael D. Stone, Laura R. McCabe, Eddie C.Y. Wang, Anwen S. Williams

**Affiliations:** a Cardiff Institute of Infection and Immunity, School of Medicine, Cardiff University, Cardiff, United Kingdom; b University Hospital Llandough, Cardiff & Vale University Health Board, Cardiff, United Kingdom; c Department of Physiology, Michigan State University, East Lansing, MI, USA; d Department of Radiology, Michigan State University, East Lansing, MI, USA; e Biomedical Imaging Research Centre, Michigan State University, East Lansing, MI, USA

**Keywords:** TNFRSF25, TNFSF15, Osteoclast, Collagen-induced arthritis, Osteoporosis, CCL3, MMP-9

## Abstract

Reduced bone density and secondary osteoporosis, resulting in increased risk of fracture, is a significant complicating factor in the inflammatory arthritides. While the exact etiology of systemic bone loss is not fully elucidated, recent insights into the tumor necrosis factor super family (TNFSF) revealed a potential role for death receptor 3 (DR3/TNFRSF25) and one of its ligands, TNF-like protein 1A (TL1A/TNFSF15). The mechanisms by which DR3/TL1A signalling modulates bone loss are unclear. We investigated the effect of DR3/TL1A signalling upon osteoclast-dependent chemokine and MMP production to unravel novel mechanisms whereby this pathway regulates OC formation and OC-dependent bone resorption. Collagen induced arthritis (CIA) was established in DR3^wt^ and DR3^ko^ mice, joints were sectioned and analysed histologically for bone damage while systemic trabecular bone loss distal to the affected joints was compared by micro-CT. Ablation of *DR3* protected DBA/1 mice against the development and progression of CIA. In DR3^ko^, joints of the ankle and mid-foot were almost free of bone erosions and long bones of mice with CIA were protected against systemic trabecular bone loss. *In vitro*, expression of DR3 was confirmed on primary human CD14^+^ osteoclast precursors by flow cytometry. These cells were treated with TL1A in osteoclast differentiation medium and TRAP^+^ osteoclasts, bone resorption, levels of osteoclast-associated chemokines (CCL3, CCL2 and CXCL8) and MMP-9 measured. TL1A intensified human osteoclast differentiation and bone resorption and increased osteoclast-associated production of CCL3 and MMP-9. Our data reveals the DR3 pathway as an attractive therapeutic target to combat adverse bone pathology associated with inflammatory arthritis. We demonstrate that DR3 is critical in the pathogenesis of murine CIA and associated secondary osteoporosis. Furthermore, we identify a novel mechanism by which the DR3/TL1A pathway directly enhances human OC formation and resorptive activity, controlling expression and activation of CCL3 and MMP-9.

## Highlights

• Joint pathology of CIA, a murine model of rheumatoid arthritis, described for the first time in the DBA DR3^ko^ mouse
• Absence of DR3 not only protects against focal bone erosion but also abrogates CIA-induced distal systemic bone loss
• DR3 expression identified for the first time on human CD14^+^ osteoclast precursors and differentiating osteoclasts
• TL1A, a DR3 ligand, concentration-dependently increases RANKL-induced osteoclast formation and resorption *in vitro*
• TL1A concentration-dependently increases RANKL-induced expression of the chemokine CCL3 and the gelatinase MMP-9 *in vitro*

## 1 Introduction

Increased prevalence of osteoporosis and decreased systemic bone mineral density at areas distal from affected joints are complicating factors for patients diagnosed with several forms of inflammatory arthritis (*e.g.* rheumatoid arthritis (RA), ankylosing spondylitis (AS) and psoriatic arthritis (PsA)) as it can lead to significant increased risk of fracture [1], [2], [3], [4]. The systemic inflammatory nature of these diseases is a substantial contributory factor to bone loss [5]. Evidence from clinical studies using biologics and non-steroidal anti-inflammatory drugs (NSAIDs) advocate the key role of cytokines (*e.g.* TNF, IL-1β, IL-6) and PGE_2_ in orchestrating inflammation-associated bone damage [6], [7], [8], [9]. However, inhibition or neutralization of these specific factors in patients does not fully ameliorate the pathology associated with inflammatory arthritis, as determined by the American College of Rheumatology improvement criteria (ACR20) [10], [11]. This suggests that unresolved progressive bone disease observed during inflammatory arthritis may well be attributed to additional factors. Members of the tumor necrosis factor superfamily (TNFSF) such as LIGHT (TNFSF14), B lymphocyte stimulator (BLyS; TNFSF13B), a proliferation-inducing ligand (APRIL; TNFSF13A) and TNF-like protein 1A (TL1A; TNFSF15) are elevated in the serum and/or synovial fluid of RA patients, potentially contributing to the bone pathology associated with the musculoskeletal disease [12], [13], [14]. LIGHT, BLyS and APRIL are capable of signalling through numerous transmembrane TNF receptor superfamily (TNFRSF) members to induce their effect. In contrast, TL1A has only been confirmed to bind to one transmembrane receptor, death receptor 3 (DR3; TNFRSF25) [15]. This study focused on the role of DR3 in mediating pathological bone loss.

Death receptor 3 (DR3; TNFRSF25, TRAMP, LARD, Apo3 and Wsl1) is involved in the pathogenesis of multiple inflammatory conditions such as inflammatory bowel disease, atherosclerosis, allergic lung inflammation and RA [14], [16], [17], [18], [19], [20]. To date DR3 has two confirmed ligands, the aforementioned TL1A, and progranulin (PGRN)/Atsttrin; which has been demonstrated to inhibit TL1A activity [21]. While little is currently known about the role of PGRN in DR3 modulation of bone more is known about DR3/TL1A signalling. The potentially important role for the DR3/TL1A pathway in regulating osteoclast (OC) formation and resorptive activity has been evidenced in murine and human studies. Cartilage damage and focal bone erosion were reduced or inhibited by DR3 or TL1A gene ablation in the murine models, antigen-induced arthritis (AIA) and collagen-induced arthritis [22], [23], [24]. Furthermore, addition of TL1A to human peripheral blood mononuclear cell (PBMC) cultures in the presence of macrophage colony stimulating factor (MCSF) and receptor activator of nuclear factor kappa B ligand (RANKL) enhanced OC formation [22]. These data imply an important role of DR3/TL1A in modulating osteoclastogenesis and bone resorption, however, the underlying mechanisms are unclear.

Signalling through the DR3/TL1A pathway following IFNγ priming on human PBMC-derived CD14^+^ macrophages and the monocytic THP-1 cell line induced expression of the chemokines CCL2 and CXCL8 [25], [26]. The function of these chemokines is not limited to cell trafficking as they also act as key mediators in the mobilisation of OC precursors and OC differentiation. Indeed, elevated expression of the chemokines CCL2, CCL3 and CXCL8 have been described in the serum and synovial fluid of RA patients while *in vitro* they have been demonstrated to enhance RANKL-induced OC formation [27], [28], [29], [30], [31], [32]. These chemokines however, have not previously been linked with DR3/TL1A dependent osteoclast-associated bone damage. While chemokines play a role in OC formation they are not directly involved in osteoclastic bone resorption. Dissolution of calcium by acidification of the resorption lacunae and proteolysis of the organic matrix by matrix metalloproteinases (MMPs), the cysteine proteinase cathepsin K and the metalloenzyme tartrate resistant acid phosphatase (TRAP) results in bone degradation [33], [34], [35]. MMP-9 specifically, is implicated in osteoclast bone resorption [35], [36]. Increased expression of MMP-9 has also been described in RA patient serum and is correlated with the collagen degradation marker hydroxyproline (OHPro), furthermore MMP-9 expression was reduced in DR3^ko^ joints undergoing AIA [24]. These initial findings suggest that CCL2, CXCL8 and MMP-9 are important downstream effector molecules by which DR3/TL1A signalling drives pathologic systemic bone loss observed in the inflammatory arthritides.

In the present study we demonstrate a critical role for DR3 in the pathogenesis of joint erosions in murine CIA and additionally reveal that DR3 drives secondary osteoporosis at sites distal from the affected small joints, using DBA/1 mice lacking the DR3 gene (DR3^ko^). *In vitro*, we identify for the first time that DR3 is expressed on human CD14^+^ OC precursors and differentiating OC. We report that TL1A directly increases OC differentiation and resorptive activity in a concentration-dependent fashion and that this is not through CCL2, but *via* elevated expression of the osteoclastogenic chemokine CCL3 and enhanced activation of the gelatinase MMP-9.

## 2 Materials and methods

### 2.1 Animals and CIA

All animal experiments were undertaken in 6–8 week-old male DBA/1 DR3^wt^ and DR3^ko^ mice [37]. Procedures were performed in accordance with Home Office-approved licenses PPL 30/2361 and 30/2928 and complied with ARRIVE guidelines. Induction of CIA, assessment of severity (paw scores) and inflammatory parameters (arthritis index) were carried out as previously described [24], [38], [39] (see supplementary methods).

### 2.2 Histological assessment of CIA joints

Architectural changes and inflammatory parameters caused by CIA were assessed histologically in the left hind leg (DR3^wt^ baseline *n* = 5, DR3^wt^ CIA *n* = 15 and DR3^ko^ baseline *n* = 5, DR3^ko^ CIA *n* = 8) as previously described to assign an arthritis index score [38], [39] (see supplementary methods).

### 2.3 Micro-computed tomography (μCT) analysis

In separate experiments, the right hind limbs (DR3^wt^ baseline *n* = 5, DR3^wt^ CIA *n* = 14 and DR3^ko^ baseline *n* = 5, DR3^ko^ CIA *n* = 8) were scanned using a GE Explore Locus microcomputed tomography (μCT) system at 20 μm voxel resolution obtained from 720 views. Beam angle of increment was 0.5; beam strength 80 peak-kV and 450 μA. Each blind run included DR3^wt^ and DR3^ko^ baseline and CIA bones, and a calibration phantom to standardize grayscale values and maintain consistency. Femoral bone analyses were performed in trabecular bone (1% of total length proximal to the growth plate, extending 2 mm toward the diaphysis, excluding outer cortical bone). Trabecular bone mineral content (BMC), bone volume/total volume (BV/TV), trabecular thickness (Tb·Th), spacing (Tb·Sp), and number (Tb·N) values were computed by MicroView software (GE). Cortical measurements were performed in a 2 × 2 × 2 mm cube midway down the bone.

### 2.4 Human osteoclastogenesis assays and analysis of resorption pits

Ethical approval was obtained from the Medical/Dental School Research Ethics Committee (09/21, Cardiff University, UK). Human peripheral blood mononuclear cells were isolated from healthy female volunteers (*n* = 7) by density gradient centrifugation using Histopaque-1077 (Sigma), and CD14^+^ monocytes isolated by magnetic cell sorting following manufacturer's instructions (Miltenyi Biotec, UK). Cells (6.4 × 10^4^; > 95% CD14^+^ purity) were added to ivory discs in RPMI supplemented with 10% FCS, 20 mM l-glutamine and 50 μg/ml Penicillin/Streptomycin (RPMI-10). After 2 h at 37 °C 5% CO_2_, discs were transferred to 48-well plates and RPMI-10 with MCSF (5 ng/ml) added. Media was replenished after 3 days and cells stained for DR3 after 7 days (classified as day 0 for OC assays). Media was replenished every 3–4 days using RPMI-10 supplemented with MCSF (5 ng/ml), RANKL (5 ng/ml), anti-polyHistidine (2.5 μg/ml) ± TL1A (10 or 100 ng/ml), all from R&D systems. Supernatants were stored at − 80 °C for further analysis. 3 discs per condition were stained for TRAP on days 7, 10 and 14. Images of five random areas of the discs were taken at × 10 magnification using a BX41 microscope and Camedia C-3030 camera (Olympus, UK) and cropped to represent 1000 μm^2^ (Corel Paint Shop Pro, Corel, UK). The number of TRAP-positive multinucleated cells and TRAP-negative/positive mononucleated cells were counted and results reported per disc. For resorption pits, discs minus cells and hematoxylin, were stained with toluidine, photographed and analysed as above (see supplementary methods).

### 2.5 Flow cytometry

Isolated human peripheral blood CD14^+^ monocytes (*n* = 7) and cells isolated from OC cultures (*n* = 1 per time-point) were stained with anti-DR3-PE (clone JD3) and anti-CD14-FITC (clone 61D3) (eBioscience). Data were acquired on an Accuri C6 flow cytometer and analysed with CFlow software (BD Biosciences).

### 2.6 Murine CIA serum and human osteoclastogenesis cell culture supernatant analysis

DR3^wt^ and DR3^ko^ CIA serum levels of IFNγ, TNFα, IL-2, IL-4, IL-5, IL-6, IL-9, IL-10, IL-13, IL-17A, IL-17F, IL-21 and IL-22 were analysed using LEGENDplex Mouse Th Cytokine Panel (BioLegend, San Diego, CA) according to the manufacturer's instructions. Levels of CCL2, CCL3, CXCL8 and total MMP-9 in human osteoclastogenesis assay supernatants were measured using DuoSet kits (R&D Systems). TNFα was measured in human osteoclastogenesis assay supernatants using an OptEIA kit (BD Biosciences).

### 2.7 Active MMP-9 zymogram

MMP-9 activity was determined by gelatine zymography following manufacturer's instructions (Bio-rad) (see supplementary methods). *In vivo* FX Pro and Molecular Imaging Software (Carestream) were used to scan gels and visualize bands.

### 2.8 Statistical analysis

Data are expressed as mean ± SEM, and statistical analysis (Mann-Whitney *U* Tests for non-parametric, unpaired *t*-Tests for parametric data, 1-way or 2-way analysis of variance (ANOVA)) performed using GraphPad Prism software (GraphPad, San Diego, USA). *p*-Values of ≤ 0.05 were considered significant and *p*-values of ≤ 0.01 highly significant.

## 3 Results

### 3.1 DR3^ko^ mice are protected against focal bone erosion in response to collagen-induced arthritis

**Fig. 1:**
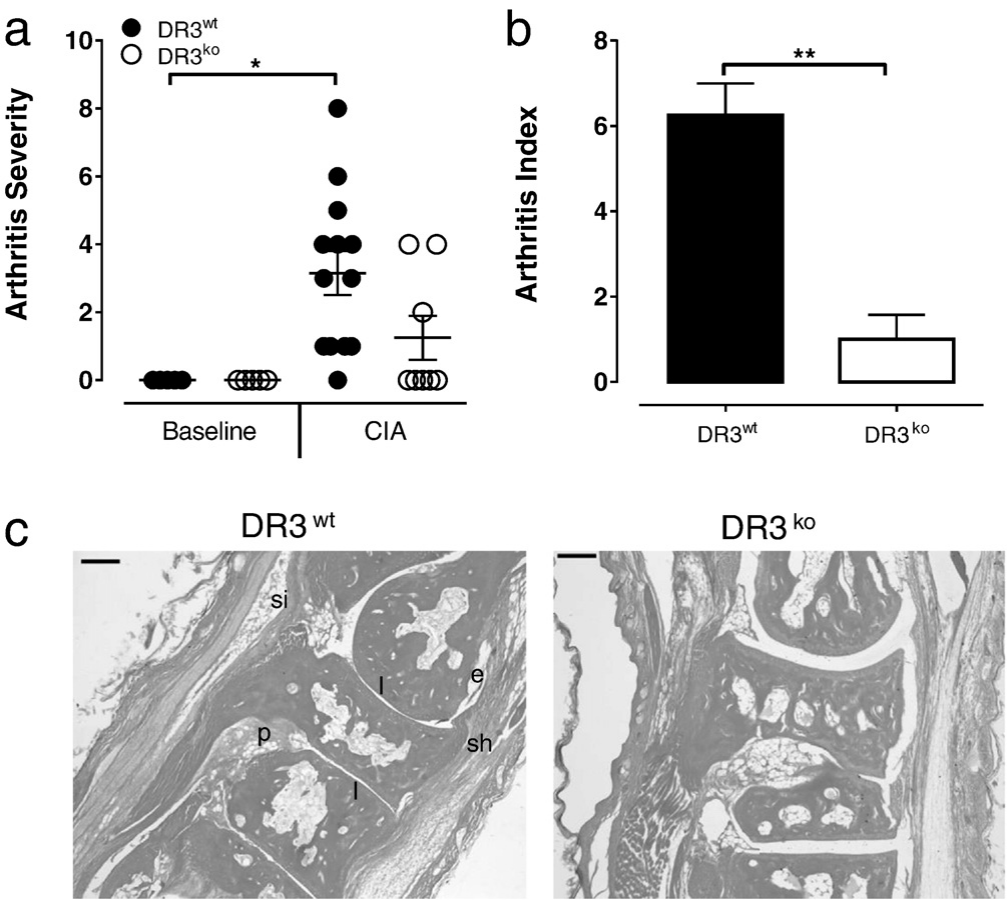
Absence of DR3 protects against collagen-induced arthritis. CIA was induced in male DR3^wt^ and DR3^ko^ mice bred onto a DBA/1 background. a) Arthritis severity was determined by level of paw swelling. DR3^wt^ CIA mice had significantly worse arthritis severity (*p* < 0.05) compared to the DR3^wt^ baseline control. No significant difference was observed between the DR3^ko^ CIA and DR3^ko^ baseline controls. b) Summary data of pathology by an arthritis index determined by scoring for erosion, cellular infiltrates, exudate and hyperplasia. Arthritis index was significantly lower in DR3^ko^ CIA compared to DR3^wt^ CIA mice (*p* < 0.01); *n* = 5–15 mice per group. c) Representative micrographs of joints from DR3^wt^ and DR3^ko^ mice following CIA induction: e = erosion; p = pannus formation; sh = synovial hyperplasia; si = synovial infiltration; l = loss of joint space. Scale bar = 0.1 mm. Statistical analysis performed with unpaired students *t*-test.

Ablation of TL1A in a model of CIA has previously been demonstrated to partially block inflammation and focal bone erosion associated with the disease pathology [23]. The use of DR3^ko^ mice to investigate the pathogenesis of inflammation and focal bone erosion in CIA however, has never been carried out. This is increasingly relevant as novel non-TNFSF ligands for DR3 that impact on TL1A signalling, such as PGRN, have recently emerged [21]. Significant increases in arthritis severity, as determined by joint swelling and number of affected joints, were observed in the DR3^wt^ CIA cohort compared to DR3^wt^ baseline controls (*p* < 0.05). In contrast, arthritis severity in the DR3^ko^ CIA cohort did not differ significantly from the DR3^ko^ controls (Fig. 1a). Histological analysis (arthritis index) of hind limbs at experimental endpoint further confirmed the importance of DR3 in CIA pathology. While the DR3^wt^ CIA mice exhibited a significantly higher arthritis index compared to the DR3^wt^ baseline (*p* < 0.001), there was no significant difference observed in arthritis index between the DR3^ko^ CIA mice compared to baseline mice. More importantly, a significantly lower arthritis index in DR3^ko^ CIA compared to DR3^wt^ CIA mice (*p* < 0.01) was observed (Fig. 1b, c). Analysis of cytokine expression in the serum of DR3^wt^ and DR3^ko^ CIA mice identified no significant difference in levels of IFNγ, TNFα or IL-6 (supplementary data Fig. 1). All other cytokines investigated were below the limit of detection.

### 3.2 Absence of DR3 protects against systemic trabecular bone loss in the distal femur during collagen-induced arthritis

**Fig. 2:**
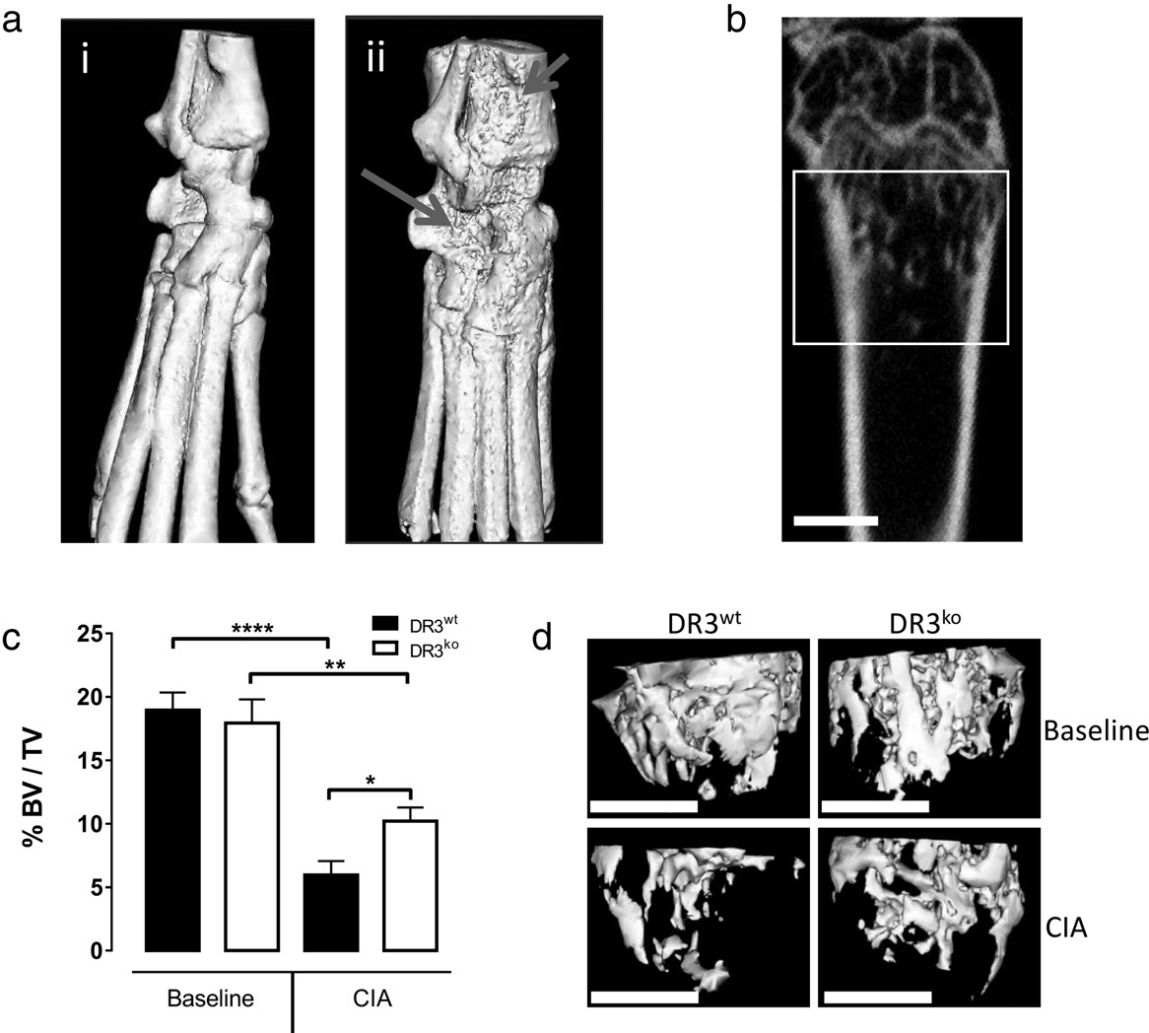
Absence of DR3 protects against systemic bone loss associated with collagen-induced arthritis. CIA was induced in male DR3^wt^ and DR3^ko^ mice. Representative images of a. i) control and ii) severe arthritis ankle joints showing erosions. Arrows indicate areas of erosion. b) Representative image of distal femoral trabecular region analysed by μCT. c) Distal femoral trabecular BV/TV was determined by μCT. Both DR3^wt^ (*p* < 0.0001) and DR3^ko^ (*p* < 0.01) CIA mice significantly lost trabecular bone compared to relevant baseline control. DR3^wt^ CIA mice lost significantly more trabecular bone than DR3^ko^ counterparts (*p* < 0.05). d) Representative μCT isosurface images. Scale bar = 1 mm. *n* = 5–14 mice per group. Statistical analysis performed with 1-Way ANOVA and Holm-Šídák post-test.

Generalized bone loss is a complicating factor in the pathogenesis of inflammatory arthritis leading to increased risk of fracture [1], [4]. We investigated whether absence of DR3 signalling could protect against systemic trabecular bone loss, distal from affected small joints, in the murine CIA model. As a measure of bone health, μCT was used to analyse the distal femur. Analysis of cortical bone parameters (Table 1) did not identify any significant differences between baseline and CIA cohorts. In contrast, the metaphyseal trabecular region displayed a significant decrease in bone volume fraction (BV/TV) in both CIA DR3^wt^ (*p* < 0.0001) and CIA DR3^ko^ (*p* < 0.01) mice compared to their baseline controls (Fig. 2c). However, the DR3^ko^ CIA mice had significantly greater trabecular BV/TV than DR3^wt^ CIA mice (DR3^wt^ = 6.0 ± 1.1%, DR3^ko^ = 10.2 ± 1.1%; *p* < 0.05). Similarly, CIA significantly decreased trabecular number (Tb·N) in both DR3^wt^ (*p* < 0.0001) and DR3^ko^ (*p* < 0.05) mice, but the decrease was greater in DR3^wt^ CIA compared to DR3^wt^ controls (Table 1). Trabecular thickness (Tb·Th, *p* < 0.0001) was only significantly decreased by CIA in DR3^wt^ but not DR3^ko^ mice. DR3^ko^ CIA mice showed significantly increased Tb·N (*p* < 0.05) and decreased Tb·Sp (*p* < 0.05) compared to DR3^wt^ CIA animals.

### 3.3 Expression of DR3 is induced by MCSF and the presence of a bone substrate on human CD14^+^ osteoclast precursors

**Fig. 3:**
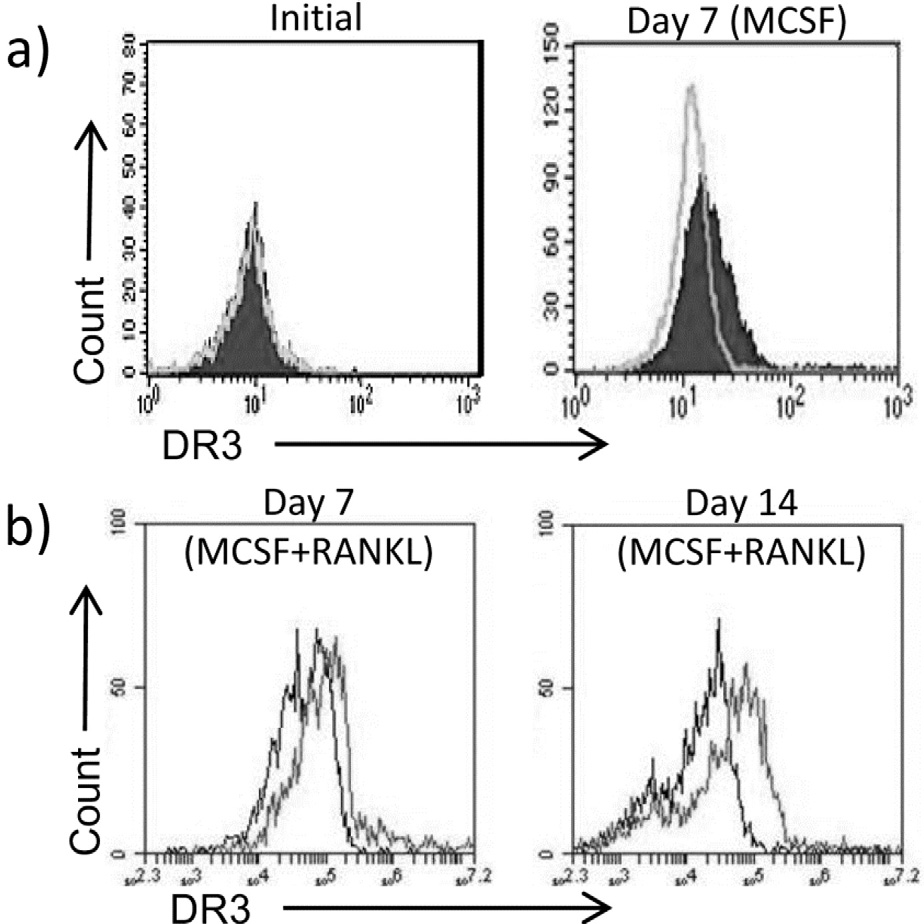
Expression of DR3 on human CD14^+^ monocyte osteoclast precursors. CD14^+^ monocytes were isolated from pre-menopausal females (*n* = 7) and cultured on ivory discs for 7 days in media + MCSF. DR3 expression was determined by flow cytometry. a) DR3 was not detected on freshly isolated CD14^+^ monocytes but confirmed after 7 days culture in MCSF (light grey line = isotype, shaded peak = DR3). b) DR3 expression was maintained in cultures following addition of RANKL (black line = isotype, grey line = DR3).

Ablation of DR3 signalling protected the ankle against CIA-induced focal bone erosion and the distal femur from systemic bone loss, implying that the DR3/TL1A pathway in mice promotes OC-dependent bone pathology during inflammatory arthritis. Addition of TL1A to osteoclastogenic human PBMC cultures has also been demonstrated to enhance OC formation [22]. However, it is unclear from these results whether the action of TL1A is through an indirect mechanism, *via* CD4^+^ T cells, or a direct effect on the OC precursors. To determine if TL1A can directly signal to human OC precursors, expression of DR3 was examined on isolated human peripheral blood CD14^+^ monocytes by flow cytometry. Samples were derived from females as osteoporosis and RA are more prevalent in women [40], [41]. While DR3 expression was not detected on the surface of freshly isolated CD14^+^ monocytes, it was up-regulated following culture for 7 days in MCSF on ivory discs indicating that TL1A could directly signal to the expanded population of osteoclast precursors (Fig. 3a). Expression of DR3 was maintained in osteoclast-containing cultures throughout the period of differentiation by RANKL and MCSF (Fig. 3b). DR3 expression was not induced on cells cultured on a glass substrate (supplementary data Fig. 2)

### 3.4 TL1A directly increases osteoclast formation from human CD14^+^ osteoclast precursors and upregulates osteoclast resorption in a concentration-dependent manner

**Fig. 4:**
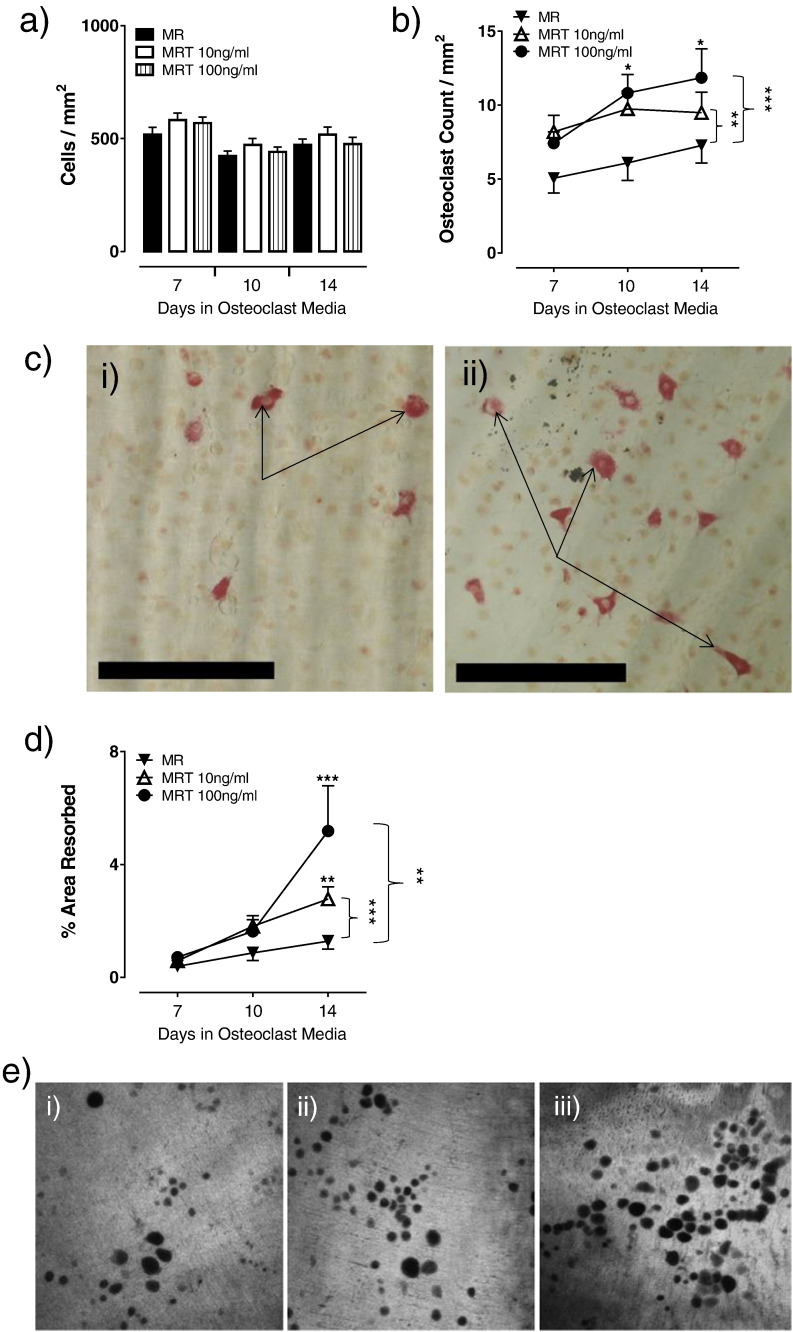
TL1A concentration-dependently increases osteoclast formation and osteoclast resorption. CD14^+^ monocytes were isolated from pre-menopausal females (*n* = 7) and cultured on ivory discs for 7 days in media + MCSF. Cells were differentiated for 7, 10 or 14 days in the presence of MCSF (M) and RANKL (R) ± TL1A (T) at 10 ng/ml or 100 ng/ml. At experiment end-point cells were stained for TRAP. a) TL1A had no effect on total cell number. b) A significant increase in OC numbers was observed in the 10 ng/ml (*p* < 0.01) and the 100 ng/ml (*p* < 0.0001) TL1A cultures across the time-course. c) Representative images of TRAP stained i) control and ii) 100 ng/ml TL1A cultures at day 14. Scale bar = 250 μm. Arrows indicate multinucleated OC. d) Discs were stained with toluidine blue and % area resorbed calculated. Significantly increased resorption was observed in the 10 ng/ml (*p* < 0.0001) and 100 ng/ml (*p* *<* 0.01) TL1A cultures across the time-course compared to control. e) Representative day 14 confocal images of i) control, ii) 10 ng/ml and iii) 100 ng/ml TL1A culture resorption pits. Statistical analysis performed with 2-Way ANOVA and Bonferroni post-test.

Having demonstrated that human CD14^+^ OC precursors express DR3 we next sought to determine the direct effect of TL1A signalling on CD14^+^ precursor OC formation and OC resorptive activity. To assess the impact of this, osteoclastogenesis assays using purified CD14^+^ monocytes on ivory discs were carried out and the number of total cells (TRAP^−^ and TRAP^+^) and TRAP^+^ multinucleated cells per mm^2^ counted. At day 7, total cell numbers in control cultures were 518 ± 33, comparable to those with 10 ng/ml (582 ± 31) and 100 ng/ml TL1A (569 ± 26) and did not change significantly throughout the time-course (Fig. 4a). In contrast, addition of TL1A had a significant concentration-dependent effect on OC formation as did time, however no interaction between the two was observed (2-way ANOVA: Time *p* < 0.05, TL1A *p* < 0.001). At days 10 and 14, OC numbers were marginally increased in the 10 ng/ml (10 ± 1 and 11 ± 1, respectively) and significantly in the 100 ng/ml TL1A cultures (9 ± 1 and 12 ± 2 respectively; *p* < 0.05) when compared to cultures without TL1A (8 ± 1 and 7 ± 1, respectively). This translated into a significant increase in OC numbers in both the 10 ng/ml (*p* < 0.01) and 100 ng/ml TL1A cultures across the time-course (*p* < 0.001; Fig. 4b, c). In the absence of RANKL, TL1A had no effect on total cell numbers and was unable to induce osteoclast formation (supplementary data Fig. 3).

To determine the functional consequences of increased OC differentiation, the % area resorbed by OC of the ivory discs was measured. At day 10 in cultures without TL1A, resorption was calculated as 0.9 ± 0.3%, which increased in the 10 ng/ml (1.8 ± 0.4%) and 100 ng/ml (1.6 ± 0.4%) TL1A cultures. At day 14, significantly increased resorption was observed in 10 ng/ml (2.8 ± 0.4%; *p* < 0.01) and 100 ng/ml TL1A cultures (5.2 ± 1.6%; *p* < 0.001) compared to those without TL1A (1.3 ± 0.3%). This translated into significantly elevated resorption in the 10 ng/ml TL1A (*p* < 0.001) and 100 ng/ml TL1A (*p* < 0.01) cultures across the time-course (Fig. 4d, e).

### 3.5 TL1A signalling enhances RANKL-induced expression of the osteoclastogenic chemokine CCL3

**Fig. 5:**
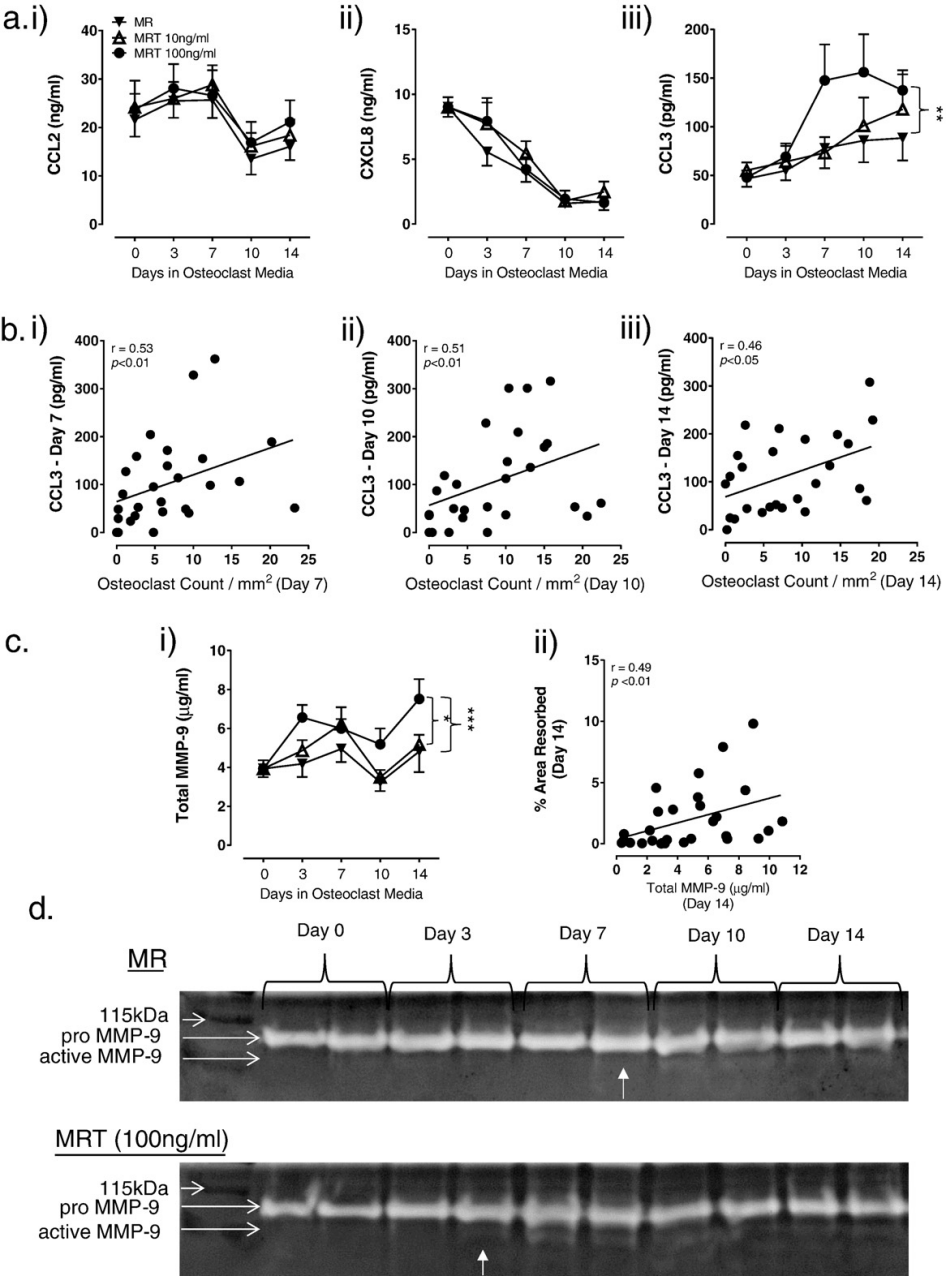
TL1A concentration-dependently increases expression of the osteoclastogenic chemokine CCL3 and gelatinase MMP-9. CD14^+^ monocytes were isolated from pre-menopausal females (*n* = 7). Cells were cultured on ivory discs for 7 days in media + MCSF and further differentiated for 7, 10 or 14 days in the presence of MCSF (M) and RANKL (R) ± TL1A (T) at 10 ng/ml or 100 ng/ml. Culture supernatants were collected at indicated time-points and tested for a) i) CCL2, ii) CXCL8 and iii) CCL3. Significantly increased CCL3 levels were detected across the time-course in 100 ng/ml TL1A cultures compared to control (*p* < 0.01). No differences were observed in CCL2 and CXCL8 levels. b) CCL3 levels significantly correlated with OC numbers at (i) day 7, (ii) day 10 and (ii) day 14. c) i) Significantly increased total MMP-9 expression was observed in the 100 ng/ml TL1A cultures across the time-course compared to control (*p* < 0.0001) and 10 ng/ml TL1A cultures (*p* < 0.05). ii) Levels of total MMP-9 significantly correlated with % area resorbed at day 14. d) Levels of pro- and active-MMP-9 in cultures were determined by gelatine zymography (*n* = 4). Representative zymograms for control (MR) and 100 ng/ml TL1A (MRT) cultures. Pro-MMP-9 was detected in both cultures across the time-course. Active MMP-9 was first detected at day 3 in the 100 ng/ml TL1A OC cultures and day 7 in control cultures. Samples run in duplicate. Statistical analysis performed with 2-Way ANOVA and Spearman correlation.

The mobilisation of OC precursors to sites of resorption and subsequent formation of OC is controlled by chemokines, including CCL2, CXCL8 and CCL3 [31], [42]. Addition of TL1A had no effect on expression of CCL2 (Fig. 5a (i)) and CXCL8 (Fig. 5a (ii)) in osteoclast-containing cultures. However, TL1A had a profound effect on levels of CCL3 (2-way ANOVA: Time *p* < 0.0001, TL1A *p* < 0.05). Across the 14-day time-course a significant overall increase in CCL3 secretion was detected in 100 ng/ml TL1A treated cells compared to untreated cultures (*p* < 0.01; Fig. 5a (iii)). Expression of CCL3 had a positive and significant correlation with OC numbers at day 7 (*r* = 0.53, *p* < 0.01; Fig. 5b (i)), day 10 (*r* = 0.51, *p* < 0.01; Fig. 5b (ii)) and day 14 (*r* = 0.46, *p* < 0.05; Fig. 5b (iii)). TL1A had no effect on CCL2, CXCL8 and CCL3 expression in cultures without RANKL (supplementary data Fig. 4).

### 3.6 TL1A signalling increases RANKL-induced MMP-9 expression and brings forward detection of active MMP-9 in osteoclast cultures

MMP-9, a gelatinase expressed by OC and their precursors, cleaves collagen present in the organic matrix during osteoclastogenic bone degradation [35]. In the present study TL1A was observed to increase total MMP-9 expression (2-way ANOVA: Time *p* < 0.0001, TL1A *p* < 0.001) with a significant increase detected across the 14-day time-course in osteoclast cultures containing 100 ng/ml TL1A compared to OC cultures containing 10 ng/ml TL1A (*p* < 0.05) and cultures without TL1A (*p* < 0.001; Fig. 5c (i)). At day 14, total MMP-9 expression correlated positively with % area resorbed (*r* = 0.49, *p* < 0.01; Fig. 5c (ii)). A gelatine zymogram was used to detect active MMP-9. The cleaved active form of MMP-9 was not detected at the start of the OC cultures (day 0). From day 3 onwards active MMP-9 was detected in OC cultures containing 100 ng/ml TL1A compared to day 7 in cultures without TL1A (Fig. 5d), suggesting that increasing levels of TL1A promotes activation of MMP-9.

## 4 Discussion

Prior to this study DR3 has been shown to drive early cartilage destruction and bone pathology associated with the murine AIA model of inflammatory arthritis [22], [24]. However, this model does not recapitulate the endogenous breach of tolerance that is typical of RA pathogenesis and as such is limited in its applicability to the disease [43]. To address these shortcomings we utilized the murine model of CIA which shares many immunological and pathological similarities with RA: generation of autoantibodies toward self, breach of tolerance, symmetrical joint involvement, peripheral joints affected and systemic development of synovial hyperplasia, pannus formation and subsequent bone resorption [43], [44], [45]. In addition to DR3, TL1A, one of DR3’s two confirmed ligands, has also been demonstrated to contribute to the development and progression of focal bone erosions in murine models of inflammatory arthritis [23], while increased levels are observed in the serum and synovial fluid of patients with inflammatory arthritis [14]. These data highlight a significant role for DR3 in pathological bone loss however, the mechanism through which DR3 signalling acts upon bone-altering cells remains poorly defined. In the current study we sought to identify the role of DR3 in the CIA model of inflammatory arthritis-induced bone loss and reveal the mechanism by which DR3/TL1A signalling affected human OC formation and resorptive activity.

We demonstrate for the first time that ablation of DR3 protects against focal bone erosions in a CIA murine model of inflammatory arthritis; the gold standard *in vivo* model for RA studies. We further reveal that absence of DR3 protected against systemic trabecular bone loss, distal from the affected small joints. In an *in vitro* human system, we show DR3 expression on CD14^+^ OC precursors and differentiating OC and that TL1A can directly affect OC differentiation by increasing expression of the osteoclastogenic chemokine CCL3. TL1A also increased OC resorptive activity, associated with enhanced expression and activation of MMP-9. All these effects are TNFα-independent as no TNFα was detected in cultures (Supplementary data Fig. 5).

The pathogenesis of inflammatory arthritis leads to focal bone erosions of small joints and systemic bone loss, resulting in an increased risk of fracture [4], [46]. Prior to this study the role of DR3 in this pathology had not been fully described, as the DR3-deletion mutant was not available on the murine DBA background. We describe for the first time murine CIA in the male DR3^ko^ mouse; as the penetrance of this model is very reproducible and less inclined to variability compared to female mice [47]. Ablation of DR3 resulted in reduced arthritis severity, as determined by reduced swelling and number of affected joints when compared to the DR3^wt^. Furthermore DR3^ko^ mice were significantly protected against focal bone erosions. These results reveal a critical role for DR3 in the pathogenesis of systemic joint damage in inflammatory arthritis and support the findings observed in DR3^ko^ mice on a C57BL/6 background in the AIA inflammatory arthritis model [24]. This protection against focal bone erosion and inflammation is not due to any baseline cellular abnormality in the myeloid cells from DR3^ko^ mice. Under canonical conditions, DR3^ko^ osteoclastogenesis from bone marrow-derived macrophages has been demonstrated to be comparable to that of the DR3^wt^, while the addition of TL1A only boosted osteoclastogenesis in DR3^wt^ cultures [22]. Furthermore, studies have shown no significant difference in naïve T cell populations in the periphery or lymphoid tissue [48]. Studies investigating both the TL1A^ko^ in CIA and DR3^ko^ mouse in other inflammatory models revealed no significant differences in the capacity to differentiate Treg (FoxP3^+^), Th1 and Th17 cells in relation to wildtype animals [23], [49]. However, studies using splenic T cells from wild-type and DR3^ko^ mice identified that TL1A signalling through DR3 supported the maintenance of T cell IL-17A expression [50], the expansion of effector T cells at sites of disease [49], expansion of T cells following viral infection [51] and expansion of T cells following bacterial infection [52]. This suggests that the DR3/TL1A pathway is not required for baseline maintenance of cell profiles and number but under inflammatory conditions maintains the signal to drive their expansion. In addition to identifying a role for DR3 in regulating joint damage we revealed a novel role in systemic bone loss. Analysis of both DR3^wt^ and DR3^ko^ CIA mice showed a reduction in femoral trabecular bone compared to their non-CIA controls, however, the amount of trabecular bone loss in DR3^ko^ mice was significantly less than their DR3^wt^ counterparts. This suggests that CIA-induced trabecular bone loss is DR3-dependent, though further experiments would be required to determine the relative contribution of DR3 on myeloid, lymphoid and stromal cells. This result reveals a previously unappreciated effect of DR3 signalling in distal systemic bone loss associated with the inflammatory arthritides [1], [2], [3], [4]. Interestingly, no difference in serum cytokine expression was observed between the DR3^wt^ and DR3^ko^ CIA mice at experimental endpoint. This suggests that the protection from both focal and systemic bone loss observed in the DR3^ko^ mice is directly dependent on the ablation of DR3 signalling and is not a consequence of subsequent changes in levels of other pro-inflammatory cytokines. The importance of these findings are increased following the discovery of a second ligand for DR3, PGRN/Atsttrin, which signals negatively through DR3 inhibiting the effects of TL1A [21]. While earlier studies by Bull, Williams et al. [22] and Wang et al. [23] identified that treatment of CIA using a TL1A antagonist, or ablation of TL1A, protected against focal bone erosion it was unknown whether this was due to decreased DR3 signalling or increased DR3/PGRN signalling.

While DR3/TL1A signalling has been implicated in pathological bone loss, its mechanism of action was not certain. Bull, Williams et al. [22] showed that addition of exogenous TL1A to PBMC cultures enhanced OC formation, but it was unclear whether the effect of TL1A was directly on the OC precursors (CD14^+^ monocytes) or *via* an intermediary cell type (*e.g.* CD4^+^ T cells). An indirect effect would be in keeping with reports that TL1A enhances CD4^+^ Th17 differentiation in RA patients, maintains Th17 numbers, with IL-17 subsequently enhancing OC differentiation [50], [53], [54]. In the present study, CD14^+^ monocytes isolated from human pre-menopausal females were utilized as a source of osteoclast precursors; as females are significantly more prone to developing osteoporosis and RA [40], [41]. Furthermore, under canonical conditions pre-menopausal females have been shown to exhibit similar levels of osteoclastogenesis to matched males thus no hormonal effects were expected [55]. DR3 was not found on circulating CD14^+^ monocytes, in agreement with previous reports [25]. However, its expression was induced following culture in the presence of MCSF on an ivory substrate, but not glass. This suggests that signals supplied by the local environment and substrate are important in DR3 induction; with signalling by Ca^2 +^ through the calcium sensing receptor (CaSR) potentially key in the induction of DR3 expression on osteoclast precursors [56]. However, these data reveal that TL1A can signal directly through DR3 on CD14^+^ OC precursors.

Studies into the effect of TL1A signalling on effector T cells have demonstrated that it drives proliferation [49], [51]. Here, addition of TL1A to CD14^+^ monocyte OC cultures had no effect on precursor expansion, indicating differential effects depending on cell type. Irrespective, TL1A had a concentration-dependent effect on eventual OC numbers as measured by TRAP staining, implying action through induction of OC differentiation. This is the first time in a primary human system that TL1A has been shown to act directly on OC precursors to enhance OC formation and is consistent with effects observed in murine RAW264.7 macrophage cells [21]. In addition to increasing OC formation, in the current study TL1A was observed to have a concentration-dependent effect on resorptive activity consistent with previous reports [22], [57]. However, it was not clear from these studies whether the increase in resorptive function was a result of the increase in OC numbers or as a result of an increase in the resorptive capacity of the OC. To investigate the underlying mechanisms which resulted in the increased OC formation and resorption, culture supernatants were analysed for soluble mediators.

Osteoclast differentiation is the process by which mononuclear cells migrate and undergo fusion into the multinucleated OC and is controlled in part by chemokines. The chemokines CCL2, CXCL8 and CCL3 have all been shown to have roles in osteoclastogenesis; CCL2-deficient mice exhibit significantly reduced OC numbers while addition of exogenous CCL2, CXCL8 or CCL3 to OC cultures enhances RANKL-induced OC formation [29], [30], [31]. Interestingly, CCL3-null mice and anti-CCL2 treated rats are protected against bone damage in models of CIA, further highlighting the importance of these chemokines in OC pathology [58], [59]. In consensus with the studies by Kang et al. [25] and Su et al. [60], CCL2 production was not affected by the addition of TL1A. Contradictory to these papers however, we report that TL1A had no effect on CXCL8 expression. This difference may be due to: use of THP-1 cells; higher concentrations of TL1A and; co-stimulation with IFNγ in the Kang et al. [25] and Su et al. [60] studies. Instead, we found CCL3 expression increased in a concentration-dependent manner implying that this is a downstream target of DR3/TL1A signalling. CCL3 levels also correlated with OC numbers across the time-course, but were unaffected by the addition of TL1A alone suggesting that TL1A effects on CCL3 are RANKL-dependent. Perhaps more significantly with regards to human disease, increased concentrations of CCL3 are present in serum and synovial fluid from RA patients and in serum of PsA patients [27], [61], [62].

To determine the mechanism for the increased bone resorption the gelatinase MMP-9 was investigated. MMP-9 has been shown to have a role in bone resorption [35], [36]. Increased expression of MMP-9 has also been identified in RA patients [63] while in murine models of inflammatory arthritis a milder pathology has been observed in MMP-9 KO mice [64]. Furthermore, MMP-9 is induced by TL1A signalling in THP-1 cells [25], has been found to be reduced in DR3^ko^ joints undergoing AIA [24] and levels of the active form are reduced in DR3^ko^ osteoclast cultures (supplementary data Fig. 6), implying that MMP-9 is a key downstream target of DR3/TL1A signalling. In the present study, TL1A increased levels of total MMP-9, which correlated significantly with levels of resorption. However, for MMP-9 to be functionally active it must be cleaved into its active form [65]. TL1A brought forward the time-point at which active MMP-9 was first observed. Whether this is due to the increased amount of total MMP-9, or TL1A promoting cleavage of MMP-9 to its active form, remains to be seen. This and the fact that TL1A-dependent MMP-9 release from primary myeloid cells may require additional priming events [24], indicate that the relationship between TL1A and MMP-9 is complex and requires further investigation.

In summary, we have identified for the first time *in vitro*, that the osteoclastogenic chemokine CCL3 and the gelatinase MMP-9 are induced downstream of DR3/TL1A signalling on primary human CD14^+^ OC precursors. We show that TL1A concentration-dependently increases OC formation and resorptive activity by increasing expression of CCL3 and expression and activation of MMP-9. Combined with our *in vivo* CIA data we demonstrate the importance of DR3 signalling in the progression of pathologic OC activity associated with inflammatory arthritis-induced focal bone erosion and secondary osteoporosis.

## Authors' roles

Study design: FLC, ECYW and ASW. Study conduct: ECYW and ASW. Data collection and analysis: FLC, JOW, ACB, RKS and LJ. Data interpretation: FLC, MDS, LRM, ECYW and ASW. Manuscript preparation: FLC, ECYW and ASW. Revising manuscript content: FLC, JOW, ACB, RKS, LJ, MDS, LRM, ECYW and ASW. Approving final version of manuscript: FLC, JOW, ACB, RKS, LJ, MDS, LRM, ECYW and ASW. FLC takes responsibility for the integrity of the data analysis.

## Competing interests

None declared.

## Figures and Tables

**Table 1:** Femoral and cortical bone parameters.

Femoralparameters	Baseline		CIA	
	DR3^wt^	DR3^ko^	DR3^wt^	DR3^ko^
BV/TV	18.96 ± 1.4	17.94 ± 1.9	5.95 ± 3.6^a^	10.21 ± 1.9^ab^
Tb·N (1/mm)	4.6 ± 0.2	4.9 ± 0.4	1.96 ± 0.3^a^	3.20 ± 0.2^ab^
Tb·Th (μm)	41.2 ± 2.0	36.2 ± 1.2	29.1 ± 1.3^a^	31.6 ± 2.1
Tb·Sp (mm)	0.18 ± 0.01	0.17 ± 0.02	0.73 ± 0.14^a^	0.29 ± 0.03^b^

Cortical parameters				
Tt·Ar (mm^2^)	1.21 ± 0.01	1.24 ± 0.09	1.32 ± 0.02	1.32 ± 0.02
Ct·Ar (mm^2^)	0.80 ± 0.02	0.82 ± 0.07	0.91 ± 0.02	0.89 ± 0.03
Ma·Ar (mm^2^)	0.44 ± 0.02	0.41 ± 0.04	0.41 ± 0.01	0.42 ± 0.02
BMD (mg/cm^3^)	748 ± 64	775 ± 48	649 ± 49	685 ± 80
BMC (mg)	0.012 ± 0.001	0.014 ± 0.002	0.013 ± 0.000	0.012 ± 0.001

a - significant to base line, b - significant compared to DR3^wt^ CIA.

## Appendix A Supplementary data

Supplementary material Image 1

## References

[1] Vosse D., de Vlam K. (2009). Osteoporosis in rheumatoid arthritis and ankylosing spondylitis. Clin. Exp. Rheumatol..

[2] Maillefert J.F., Aho L.S., El Maghraoui A., Dougados M., Roux C. (2001). Changes in bone density in patients with ankylosing spondylitis: a two-year follow-up study. Osteoporos. Int..

[3] Frediani B., Allegri A., Falsetti P., Storri L., Bisogno S., Baldi F. (2001). Bone mineral density in patients with psoriatic arthritis. J. Rheumatol..

[4] Weiss R.J., Wick M.C., Ackermann P.W., Montgomery S.M. (2010). Increased fracture risk in patients with rheumatic disorders and other inflammatory diseases — a case-control study with 53,108 patients with fracture. J. Rheumatol..

[5] Hardy R., Cooper M.S. (2009). Bone loss in inflammatory disorders. J. Endocrinol..

[6] Maini R., Clair E.W.S., Breedveld F., Furst D., Kalden J., Weisman M. (1999). Infliximab (chimeric anti-tumour necrosis factor α monoclonal antibody) versus placebo in rheumatoid arthritis patients receiving concomitant methotrexate: a randomised phase III trial. Lancet.

[7] Cohen S.B., Moreland L.W., Cush J.J., Greenwald M.W., Block S., Shergy W.J. (2004). A multicentre, double blind, randomised, placebo controlled trial of anakinra (Kineret), a recombinant interleukin 1 receptor antagonist, in patients with rheumatoid arthritis treated with background methotrexate. Ann. Rheum. Dis..

[8] Maini R.N., Taylor P.C., Szechinski J., Pavelka K., Broll J., Balint G. (2006). Double-blind randomized controlled clinical trial of the interleukin-6 receptor antagonist, tocilizumab, in European patients with rheumatoid arthritis who had an incomplete response to methotrexate. Arthritis Rheum..

[9] Bombardier C., Laine L., Reicin A., Shapiro D., Burgos-Vargas R., Davis B. (2000). Comparison of upper gastrointestinal toxicity of rofecoxib and naproxen in patients with rheumatoid arthritis. VIGOR Study Group. N. Engl. J. Med..

[10] Felson D.T., Anderson J.J., Boers M., Bombardier C., Furst D., Goldsmith C. (1995). American College of Rheumatology preliminary definition of improvement in rheumatoid arthritis. Arthritis Rheum..

[11] Bresnihan B., Alvaro-Gracia J.M., Cobby M., Doherty M., Domljan Z., Emery P. (1998). Treatment of rheumatoid arthritis with recombinant human interleukin-1 receptor antagonist. Arthritis Rheum..

[12] Edwards J.R., Sun S.G., Locklin R., Shipman C.M., Adamopoulos I.E., Athanasou N.A. (2006). LIGHT (TNFSF14), a novel mediator of bone resorption, is elevated in rheumatoid arthritis. Arthritis Rheum..

[13] Tan S.M., Xu D., Roschke V., Perry J.W., Arkfeld D.G., Ehresmann G.R. (2003). Local production of B lymphocyte stimulator protein and APRIL in arthritic joints of patients with inflammatory arthritis. Arthritis Rheum..

[14] Bamias G., Siakavellas S.I., Stamatelopoulos K.S., Chryssochoou E., Papamichael C., Sfikakis P.P. (2008). Circulating levels of TNF-like cytokine 1A (TL1A) and its decoy receptor 3 (DcR3) in rheumatoid arthritis. Clin. Immunol..

[15] Bossen C., Ingold K., Tardivel A., Bodmer J.-L., Gaide O., Hertig S. (2006). Interactions of tumor necrosis factor (TNF) and TNF receptor family members in the mouse and human. J. Biol. Chem..

[16] Sun X., Zhao J., Liu R., Jia R., Sun L., Li X. (2013). Elevated serum and synovial fluid TNF-like ligand 1A (TL1A) is associated with autoantibody production in patients with rheumatoid arthritis. Scand. J. Rheumatol..

[17] McLaren J.E., Calder C.J., McSharry B.P., Sexton K., Salter R.C., Singh N.N. (2010). The TNF-like protein 1A-death receptor 3 pathway promotes macrophage foam cell formation in vitro. J. Immunol..

[18] Bamias G., Mishina M., Nyce M., Ross W.G., Kollias G., Rivera-Nieves J. (2006). Role of TL1A and its receptor DR3 in two models of chronic murine ileitis. Proc. Natl. Acad. Sci. U. S. A..

[19] Fang L., Adkins B., Deyev V., Podack E.R. (2008). Essential role of TNF receptor superfamily 25 (TNFRSF25) in the development of allergic lung inflammation. J. Exp. Med..

[20] Osawa K., Takami N., Shiozawa K., Hashiramoto A., Shiozawa S. (2004). Death receptor 3 (DR3) gene duplication in a chromosome region 1p36.3: gene duplication is more prevalent in rheumatoid arthritis. Genes Immun..

[21] Liu C., Li X.-X., Gao W., Liu W., Liu D.-S. (2014). Progranulin-derived Atsttrin directly binds to TNFRSF25 (DR3) and inhibits TNF-like ligand 1A (TL1A) activity. PLoS One.

[22] Bull M.J., Williams A.S., Mecklenburgh Z., Calder C.J., Twohig J.P., Elford C. (2008). The death receptor 3-TNF-like protein 1A pathway drives adverse bone pathology in inflammatory arthritis. J. Exp. Med..

[23] Wang X., Hu Y., Charpentier T., Lamarre A., Qi S., Wu J. (2013). TNF-like ligand 1A (TL1A) gene knockout leads to ameliorated collagen-induced arthritis in mice: Implication of TL1A in humoral immune responses. J. Immunol..

[24] Wang E.C.Y., Newton Z., Hayward O.A., Clark S.R., Collins F., Perks W.V. (2014). Regulation of early cartilage destruction in inflammatory arthritis by death receptor 3. Arthritis Rheumatol. (Hoboken, N.J.).

[25] Kang Y.-J., Kim W.-J., Bae H.-U., Kim D.-I., Park Y.B., Park J.-E. (2005). Involvement of TL1A and DR3 in induction of pro-inflammatory cytokines and matrix metalloproteinase-9 in atherogenesis. Cytokine.

[26] Kim S.H., Lee W.H., Kwon B.S., Oh G.T., Choi Y.H., Park J.E. (2001). Tumor necrosis factor receptor superfamily 12 may destabilize atherosclerotic plaques by inducing matrix metalloproteinases. Jpn. Circ. J..

[27] Koch A.E., Kunkel S.L., Harlow L.A., Mazarakis D.D., Haines G.K., Burdick M.D. (1994). Macrophage inflammatory protein-1 alpha. A novel chemotactic cytokine for macrophages in rheumatoid arthritis. J. Clin. Invest..

[28] Koch A.E., Kunkel S.L., Harlow L.A., Johnson B., Evanoff H.L., Haines G.K. (1992). Enhanced production of monocyte chemoattractant protein-1 in rheumatoid arthritis. J. Clin. Invest..

[29] Miyamoto K., Ninomiya K., Sonoda K.-H., Miyauchi Y., Hoshi H., Iwasaki R. (2009). MCP-1 expressed by osteoclasts stimulates osteoclastogenesis in an autocrine/paracrine manner. Biochem. Biophys. Res. Commun..

[30] Watanabe T., Kukita T., Kukita A., Wada N., Toh K., Nagata K. (2004). Direct stimulation of osteoclastogenesis by MIP-1alpha: evidence obtained from studies using RAW264 cell clone highly responsive to RANKL. J. Endocrinol..

[31] Bendre M.S., Montague D.C., Peery T., Akel N.S., Gaddy D., Suva L.J. (2003). Interleukin-8 stimulation of osteoclastogenesis and bone resorption is a mechanism for the increased osteolysis of metastatic bone disease. Bone.

[32] Feldmann M., Brennan F.M., Maini R.N. (1996). Role of cytokines in rheumatoid arthritis. Annu. Rev. Immunol..

[33] Kamiya T., Kobayashi Y., Kanaoka K., Nakashima T., Kato Y., Mizuno A. (1998). Fluorescence microscopic demonstration of cathepsin K activity as the major lysosomal cysteine proteinase in osteoclasts. J. Biochem..

[34] Teitelbaum S.L. (2000). Bone resorption by osteoclasts. Science.

[35] Everts V., Delaissé J.M., Korper W., Beertsen W. (1998). Cysteine proteinases and matrix metalloproteinases play distinct roles in the subosteoclastic resorption zone. J. Bone Miner. Res..

[36] Grassi F., Cristino S., Toneguzzi S., Piacentini A., Facchini A., Lisignoli G. (2004). CXCL12 chemokine up-regulates bone resorption and MMP-9 release by human osteoclasts: CXCL12 levels are increased in synovial and bone tissue of rheumatoid arthritis patients. J. Cell. Physiol..

[37] Collins F.L., Williams J.O., Bloom A.C., Stone M.D., Choy E., Wang E.C.Y. (2015). Death receptor 3 (TNFRSF25) increases mineral apposition by osteoblasts and region specific new bone formation in the axial skeleton of male DBA/1 mice. J. Immunol. Res..

[38] Evans L., Williams A.S., Hayes A.J., Jones S.A., Nowell M. (2011). Suppression of leukocyte infiltration and cartilage degradation by selective inhibition of pre-B cell colony-enhancing factor/visfatin/nicotinamide phosphoribosyltransferase: Apo866-mediated therapy in human fibroblasts and murine collagen-induced arthrit. Arthritis Rheum..

[39] Reynolds S.L., Williams A.S., Williams H., Smale S., Stephenson H.J., Amos N. (2012). Contractile, but not endothelial, dysfunction in early inflammatory arthritis: a possible role for matrix metalloproteinase-9. Br. J. Pharmacol..

[40] van Vollenhoven R.F. (2009). Sex differences in rheumatoid arthritis: more than meets the eye…. BMC Med..

[41] Melton L.J. (2001). The prevalence of osteoporosis: gender and racial comparison. Calcif. Tissue Int..

[42] Ishii T., Kikuta J., Kubo A., Ishii M. (2010). Control of osteoclast precursor migration: a novel point of control for osteoclastogenesis and bone homeostasis. IBMS Bonekey..

[43] Asquith D.L., Miller A.M., McInnes I.B., Liew F.Y. (2009). Animal models of rheumatoid arthritis. Eur. J. Immunol..

[44] Kannan K., Ortmann R.A., Kimpel D. (2005). Animal models of rheumatoid arthritis and their relevance to human disease. Pathophysiology.

[45] Joe B., Wilder R.L. (1999). Animal models of rheumatoid arthritis. Mol. Med. Today..

[46] Schett G., Gravallese E. (2012). Bone erosion in rheumatoid arthritis: mechanisms, diagnosis and treatment. Nat. Rev. Rheumatol..

[47] Holmdahl R., Jansson L., Andersson M. (1986). Female sex hormones suppress development of collagen-induced arthritis in mice. Arthritis Rheum..

[48] Wang E.C., Thern A., Denzel A., Kitson J., Farrow S.N., Owen M.J. (2001). DR3 regulates negative selection during thymocyte development. Mol. Cell. Biol..

[49] Meylan F., Davidson T.S., Kahle E., Kinder M., Acharya K., Jankovic D. (2008). The TNF-family receptor DR3 is essential for diverse T cell-mediated inflammatory diseases. Immunity.

[50] Jones G.W., Stumhofer J.S., Foster T., Twohig J.P., Hertzog P., Topley N. (2011). Naive and activated T cells display differential responsiveness to TL1A that affects Th17 generation, maintenance, and proliferation. FASEB J..

[51] Twohig J.P., Marsden M., Cuff S.M., Ferdinand J.R., Gallimore A.M., Perks W.V. (2012). The death receptor 3/TL1A pathway is essential for efficient development of antiviral CD4^+^ and CD8^+^ T-cell immunity. FASEB J..

[52] Buchan S.L., Taraban V.Y., Slebioda T.J., James S., Cunningham A.F., Al-Shamkhani A. (2011). Death receptor 3 is essential for generating optimal protective CD4(+) T-cell immunity against *Salmonella*. Eur. J. Immunol..

[53] Zhou M., Liu R., Su D., Feng X., Li X. (2014). TL1A increased the differentiation of peripheral Th17 in rheumatoid arthritis. Cytokine.

[54] Kotake S., Udagawa N., Takahashi N., Matsuzaki K., Itoh K., Ishiyama S. (1999). IL-17 in synovial fluids from patients with rheumatoid arthritis is a potent stimulator of osteoclastogenesis. J. Clin. Invest..

[55] Jevon M., Sabokbar A., Fujikawa Y., Hirayama T., Neale S.D., Wass J. (2002). Gender- and age-related differences in osteoclast formation from circulating precursors. J. Endocrinol..

[56] Mentaverri R., Yano S., Chattopadhyay N., Petit L., Kifor O., Kamel S. (2006). The calcium sensing receptor is directly involved in both osteoclast differentiation and apoptosis. FASEB J..

[57] Zhang J., Wang X., Fahmi H., Wojcik S., Fikes J., Yu Y. (2009). Role of TL1A in the pathogenesis of rheumatoid arthritis. J. Immunol..

[58] Ogata H., Takeya M., Yoshimura T., Takagi K., Takahashi K. (1997). The role of monocyte chemoattractant protein-1 (MCP-1) in the pathogenesis of collagen-induced arthritis in rats. J. Pathol..

[59] Chintalacharuvu S.R., Wang J.X., Giaconia J.M., Venkataraman C. (2005). An essential role for CCL3 in the development of collagen antibody-induced arthritis. Immunol. Lett..

[60] Su W.B., Chang Y.-H., Lin W.-W., Hsieh S.-L. (2006). Differential regulation of interleukin-8 gene transcription by death receptor 3 (DR3) and type I TNF receptor (TNFRI). Exp. Cell Res..

[61] Bao J., Liu W., Bao Y.-X. (2014). Clinical immunology recombinant human interleukin receptor antagonist influences serum chemokines in patients with rheumatoid arthritis. Cent. Eur. J. Immunol..

[62] Szodoray P., Alex P., Chappell-Woodward C.M., Madland T.M., Knowlton N., Dozmorov I. (2007). Circulating cytokines in Norwegian patients with psoriatic arthritis determined by a multiplex cytokine array system. Rheumatology.

[63] Ahrens D., Koch A.E., Pope R.M., Stein-Picarella M., Niedbala M.J. (1996). Expression of matrix metalloproteinase 9 (96-kd gelatinase B) in human rheumatoid arthritis. Arthritis Rheum..

[64] Itoh T., Matsuda H., Tanioka M., Kuwabara K., Itohara S., Suzuki R. (2002). The role of matrix metalloproteinase-2 and matrix metalloproteinase-9 in antibody-induced arthritis. J. Immunol..

[65] Toth M., Chvyrkova I., Bernardo M.M., Hernandez-Barrantes S., Fridman R. (2003). Pro-MMP-9 activation by the MT1-MMP/MMP-2 axis and MMP-3: role of TIMP-2 and plasma membranes. Biochem. Biophys. Res. Commun..

