# Supplementary material for: CCL3 and MMP-9 are induced by TL1A during death receptor 3 (TNFRSF25)-dependent osteoclast function and systemic bone loss
Source: Bone. 2017 Apr;97:94–104. doi: 10.1016/j.bone.2017.01.002 (PMC5378198; doi:10.1016/j.bone.2017.01.002)
Supplement: Supplementary file 1 — Supplementary material [file mmc1.doc]

**Supplementary Material**

**CCL3 and MMP-9 are Induced Downstream of Death Receptor 3 (TNFRSF25)-Dependent Osteoclast Function and Systemic Bone Loss**

**Supplementary Caption**

**Supplementary methods and materials**: Animals and CIA; Histological assessment of CIA joints; Osteoclastogenesis assays and analysis of resorption pits; Active MMP-9 zymogram

**Supplementary Data Figure 1:** Levels of Pro-Inflammatory Cytokines in DR3^wt^ and DR3^ko^ CIA Serum

**Supplementary Data Figure 2:** Expression of DR3 on CD14^+^ Monocyte Osteoclast Precursors Cultured on Glass Coverslips

**Supplementary Data Figure 3:** TL1A does not Induce CD14^+^ Monocyte Proliferation or Osteoclast Differentiation in the Absence of RANKL

**Supplementary Data Figure 4:** TL1A does not Effect CCL2, CXCL8 and CCL3 Expression in the Absence of RANKL

**Supplementary Data Figure 5:** TNF is not Expressed in Human CD14^+^ Osteoclast Cultures

**Supplementary Data Figure 6:** Active MMP-9 is Detected Earlier in DBA/1 DR3^wt^ Osteoclast Cultures

Materials and Methods

**Animals and CIA**

For induction of CIA, 2 mg/ml of chicken type II collagen (CII; Sigma-Aldrich) was emulsified with an equal volume of complete Freund’s adjuvant and 100 µl of collagen/adjuvant mixture injected intradermally into several sites near the base of the tail of 8-wk-old male DBA/1 DR3^wt^ and DR3^ko^ mice. Twenty one days after the first injection, a second identical booster was administered to each mouse. The day of the first immunization was designated as day 0. Arthritis severity was assessed daily until termination on day 28.

The severity of arthritis was assessed in each animal, in a blinded manner, using a clinical scoring system: 0 = normal; 1 = mild but definite swelling in the ankle or wrist joint or redness and swelling limited to individual digits regardless of the number of digits affected; 2 = moderate swelling of ankle or wrist; 3 = severe redness and swelling of the ankle or wrist and proximal phalangeal joints; and 4 = maximally inflamed limb with involvement of multiple joints, no ankyloses [38, 39]. Cumulative scores were assessed with a maximal score of 12 per animal. Hind paw ankle swelling was measured (in mm) using a modified POCO 2T spring caliper gauge (Kroeplin Längenmesstechnik).

**Histological assessment of CIA joints**

Left hind limb ankle joints were fixed in ethanol, decalcified with formic acid and processed through to paraffin wax blocks. Midsagittal serial sections (7 µm thick) were stained with hematoxylin and eosin. Histology sections were scored by 2 independent observers who were blinded to the genotype of the mice under investigation. An established histology scoring method was used [22, 38], in which synovial hyperplasia was scored 0–3, inflammatory cells within the synovial tissue was scored 0–5, inflammatory cells within the synovial cavity was scored 0–3, and articular cartilage/subchondral bone erosion was scored 0–3. All parameters were subsequently summed to give an arthritis index.

**Osteoclastogenesis Assays and Analysis of Resorption Pits**

For resorption pits, ivory discs were individually sonicated for 1 minute to disrupt adherent cells. Remaining cells were removed from discs by gentle rubbing with a cotton bud. 1% hydrogen peroxide solution was added to each disc and incubated overnight at room temperature with mechanical shaking to remove hematoxylin stain. For light microscopy analysis of resorption pits, discs were washed twice with dH_2_O and 0.5% toluidine blue stain added and incubated for 1 minute at room temperature. Stain was removed and discs washed twice with dH_2_O before being photographed and analysed.

**Active MMP-9 Zymogram**

Samples were diluted 1:5 in zymogram sample buffer (Bio-Rad) and added to a 10% ‘Ready gel’ zymogram gel (Bio-Rad). A pro MMP-9 control at 10 ng/ml was used as a reference. Gels were run for 2 hours at a constant 100V. After running, gels were incubated at room temperature in renaturation buffer (Bio-Rad) for 15 minutes before being transferred into development buffer (Bio-Rad) and incubated at 37°C for 20 hours. MMP activity was visualized by staining the gels with 0.25% coomassie blue stain for 30 minutes before incubating in destain (dH_2_O, methanol and acetic acid; 4.5:4.5:1) for 1 hour to remove non-specific staining. A broad range protein ladder (Bio-Rad) was used to determine the molecular weight of the bands.

Supplementary Figures


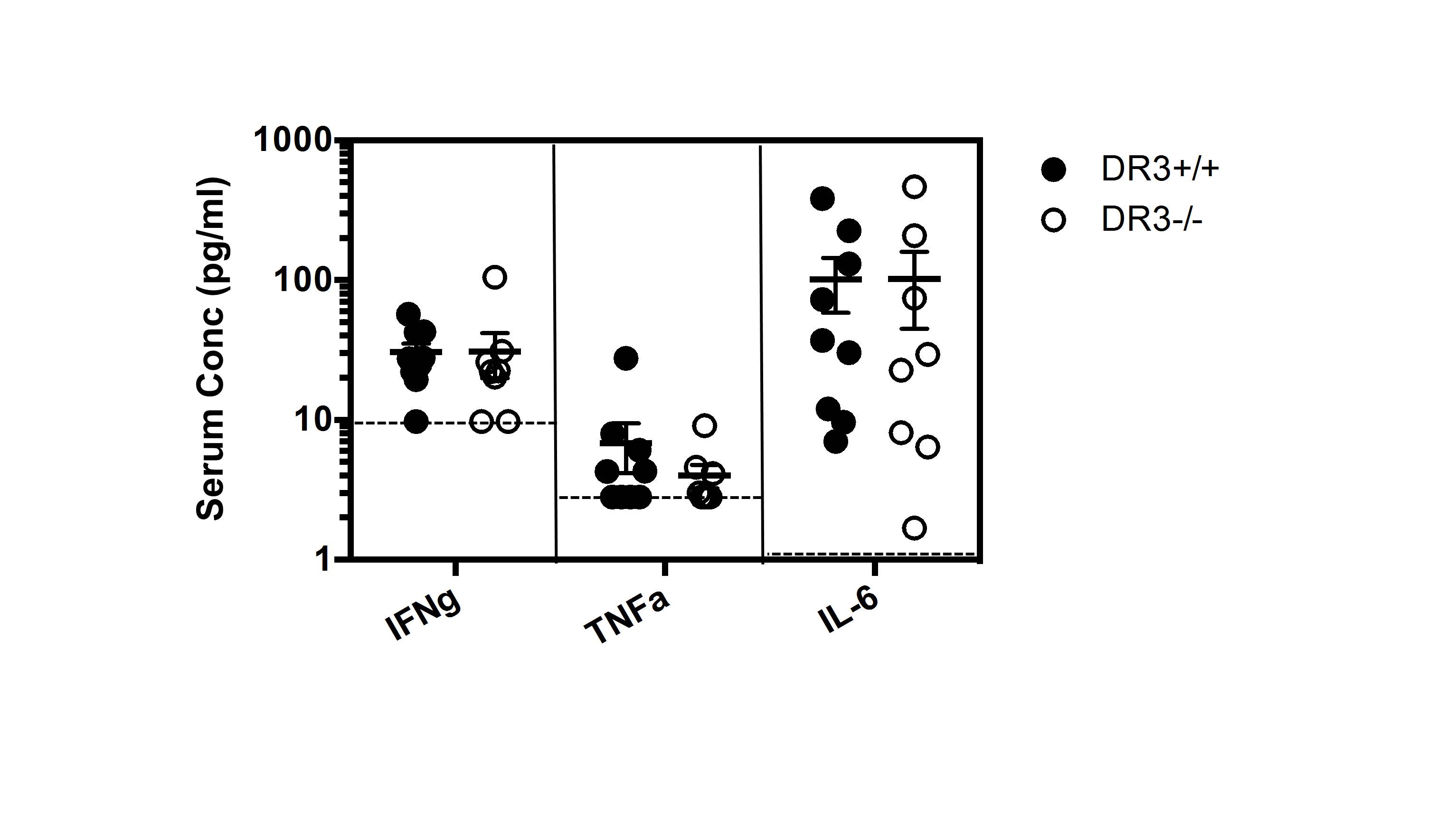


**Supplementary data Fig 1: Levels of Pro-Inflammatory Cytokines in DR3^wt^ and DR3^ko^ CIA Serum**

Serum was isolated from DR3^wt^ (n=8) and DR3^ko^ (n=8) CIA mice at experimental endpoint and analysed for cytokine expression by LEGENDplex. No significant difference in levels of IFNγ, TNFα or IL-6 was observed. All other cytokines investigated were below level of detection. Statistical analysis performed by 2-way ANOVA.

Supplementary Figures


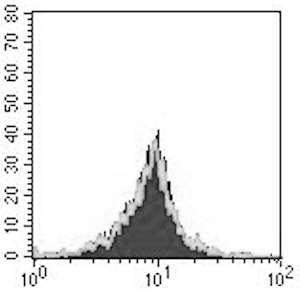


**Supplementary data Fig 2: Expression of DR3 on CD14^+^ Monocyte Osteoclast Precursors Cultured on Glass Coverslips**

CD14^+^ monocytes were isolated from pre-menopausal females (n=2) and cultured on glass coverslips for 7 days in media+MCSF. DR3 expression was determined by flow cytometry. DR3 was not detected on CD14^+^ monocytes after 7 days culture in MCSF (light grey line=isotype, shaded peak=DR3).

Supplementary Figures


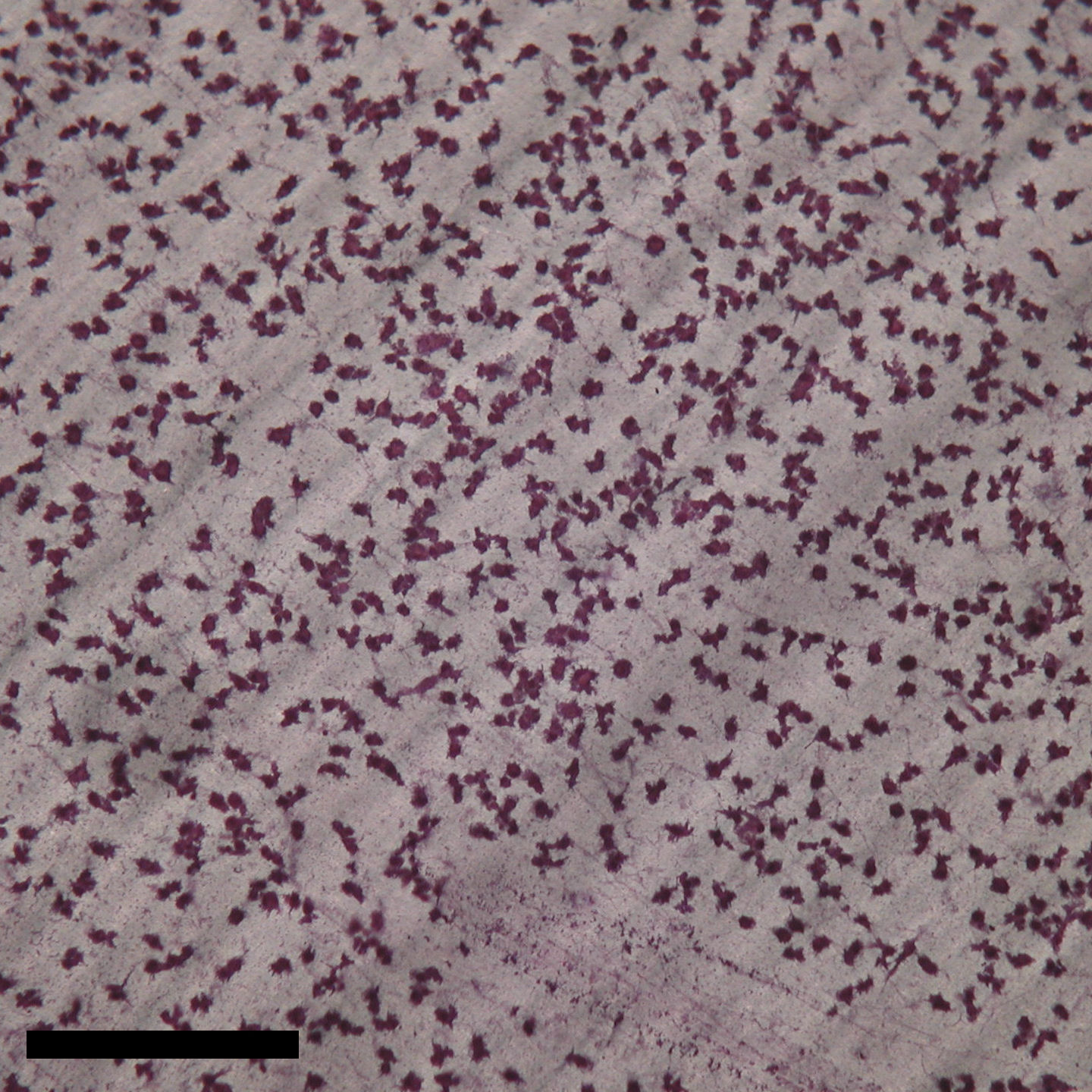

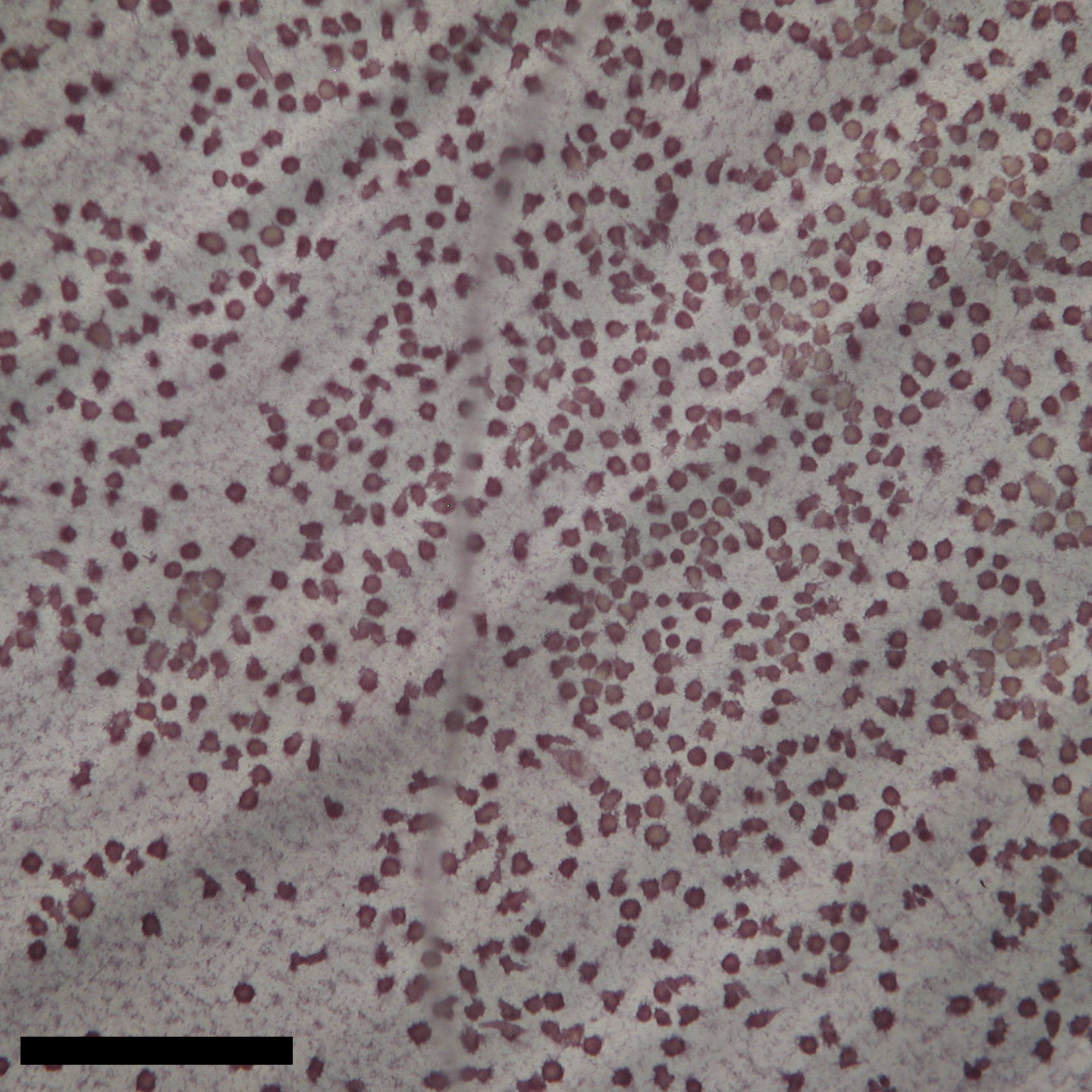

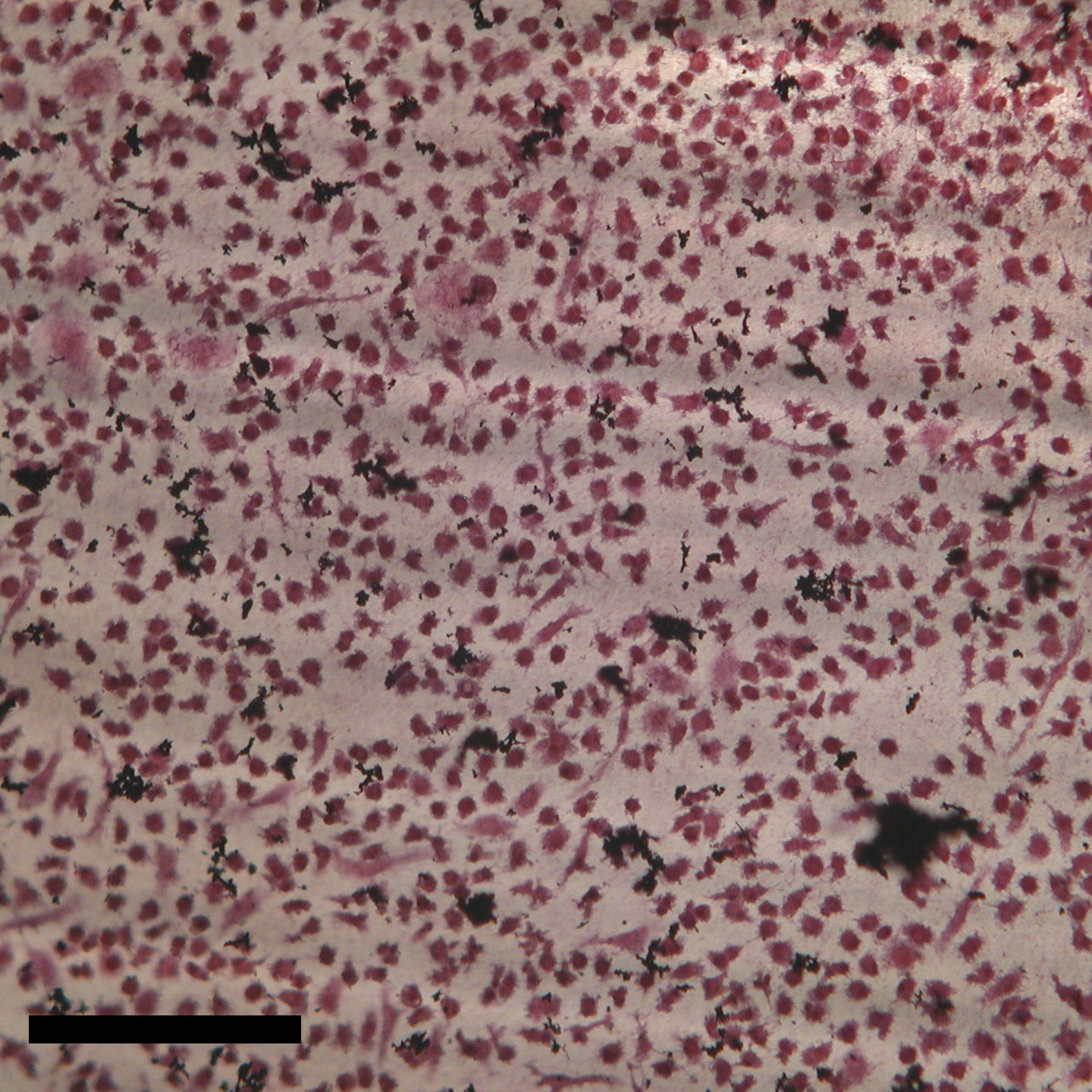


**Supplementary data Fig 3: TL1A does not Induce CD14^+^ Monocyte Proliferation or Osteoclast Differentiation in the Absence of RANKL**

CD14^+^ monocytes were isolated from pre-menopausal females (n=7) and cultured on ivory discs for 7 days in media+MCSF. Cells were differentiated for 7, 10 or 14 days in the presence of MCSF (M) ± TL1A (T) at 10ng/ml or 100ng/ml. At experiment end-points cells were stained for TRAP. TL1A had no effect on total cell number and did not induce osteoclast differentiation. Representative day 14 images of MCSF, MCSF+TL1A 10ng/ml and MCSF+TL1A 100ng/ml cultures. Scale bar=250µm. Statistical analysis performed with 2-Way ANOVA and Bonferroni post-test.

Supplementary Figures

**Supplementary data Fig 4: TL1A does not Effect CCL2, CXCL8 and CCL3 Expression in the Absence of RANKL**

CD14^+^ monocytes were isolated from pre-menopausal females (n=7). Cells were cultured on ivory discs for 7 days in media+MCSF and further differentiated for 7, 10 or 14 days in the presence of MCSF (M) ± TL1A (T) at 10ng/ml or 100ng/ml. Culture supernatants were collected at indicated time-points and tested for CCL2, CXCL8 and CCL3. TL1A had no effect on chemokine expression. Statistical analysis performed with 2-Way ANOVA.

Supplementary Figures

**Supplementary data Fig 5: TNF is not Expressed in Human CD14^+^ Osteoclast Cultures**

CD14^+^ monocytes were isolated from pre-menopausal females (n=4). Cells were cultured on ivory discs for 7 days in media+MCSF and further differentiated for 7, 10 or 14 days in the presence of MCSF (M) and RANKL (R) ± TL1A (T) at 10ng/ml or 100ng/ml. Culture supernatants were collected at indicated time-points and tested for TNFα. TNFα was not detected in any of the cultures.

Supplementary Figures

**Supplementary data Fig 6: Active MMP-9 is Detected Earlier in DBA/1 DR3^wt^ Osteoclast Cultures**

Bone marrow cells were isolated from the femora of female DBA/1 DR3^wt^ (n=6) and DR3^ko^ (n=6) and cultured for 10 days on ivory discs in the presence of MCSF and RANKL. Supernatants were collected on day 7 and day 10 and analysed for expression of pro MMP-9 and active MMP-9 by zymography. Expression of pro MMP-9 trended lower in DR3^ko^ osteoclast cultures at day 7 with no significant difference observed at day 10. Active MMP-9 was first observed at day 7 in DR3^wt^ cultures but was not observed until day 10 in DR3^ko^ osteoclast cultures where levels trended lower. Statistical analysis performed with One-way ANOVA and Bonferroni post-test.
